# Porcine health management - the pig in a central position

**DOI:** 10.1186/2055-5660-1-1

**Published:** 2015-04-16

**Authors:** Maurice B Pensaert, Guy-Pierre Martineau

**Affiliations:** 1 Laboratory of Virology, Faculty of Veterinary Medicine, University of Ghent, Ghent, Belgium; 2 French National Veterinary College, Toulouse, France

## Abstract

We are proud to launch a new open access journal, entitled Porcine Health Management (PHM). This new journal, which puts the pig in a central position, has been jointly established by the European College of Porcine Health and Management and the European Association of Porcine Health Management.

PHM is interested in publishing articles of high quality and novelty, but we also consider research results of more general interest and clinical features to the readers. We believe that putting the pig at the center of this journal is unique and will serve both our favored animal species and our professions.

We hope that you will enjoy the PHM journal and that you will consider it when deciding where to publish your work.

## Editorial

“People worry about pigs. Pigs are so compelling, so mysterious, so contradictory – finicky yet fat, massive yet dainty, stolid yet smart. We can’t decide what we think of them. We’re uneasy about our role in their life and death. We make images of them that reveal our complex feelings—our affection, our revulsion, our sentimentality, and our guilt” [1].

Harry Truman said “No man should be allowed to be president who does not understand hogs” [2].

“Dogs look up to you, cats look down on you and pigs think you’re their equal” is attributed to an old rural New England hogophile [3].

George Orwell in his book “Animal Farm” mentions the natural hierarchy that evolves when animals live together: “the work of teaching and organizing the others naturally fell to the pigs, who were recognized as being the cleverest of animals”.

In many aspects, the physiology of pigs resembles very closely that of humans, and pigs often serve as experimental models for surgery, for performing physiological studies, and also for studying pathogenesis and immunological responses to a variety of infectious organisms.

It is well known that, for ages and throughout the globe, pigs have intrigued humans and have even been a close part of their life. For this reason, the relationship between people and pigs has frequently been pictured by politicians, cartoonists, and writers [1].

It is also clear that the pig, as animal species, has been and still is being studied intensively from different angles by medical researchers, geneticists, nutritionists, and other porcine specialists. Intensification and industrialization of pig production in recent times has been challenging not only for the pig itself but also for those of us who are directly involved. Every aspect of pig keeping has become highly specialized and demanding; new diseases emerge, changes occur rapidly and are sometimes hard to follow. With this rapid evolution, the whole pig, the entire picture of pig raising, the interdisciplinary features, the interactive elements involved and their effects are often difficult to follow, and this process may even become more problematic in the future.

Let us admit, the pig is an amazing animal that has served the human population in different ways, in good and bad times and throughout history, and still continues to do so. This animal species thus asks for and deserves a science-based journal that is concerned with various aspects related to medicine and production in modern pig raising and that puts the pig centrally on the scene.

Clearly, it is rather difficult nowadays for the pig scientist involved in different pig-related subjects, for the pig veterinarian in his everyday job and for the pig manager, to keep up with all the scientific information published in different sources of the literature.

We are, therefore, proud to launch a new open access journal, entitled *Porcine Health Management* (PHM) (http://www.porcinehealthmanagement.com). This new journal, which puts the pig in a central position, has been jointly established by the European College of Porcine Health and Management (http://www.ecphm.org) and the European Association Porcine of Porcine Health Management (http://www.eaphm.org).

PHM intends to treat different topics of swine raising including: infectious and non-infectious diseases, reproduction, epidemiology, housing, nutrition, medicines, management, economics, genetics, animal welfare, ethics, legislation, and food safety. It aims to serve as an international platform for sharing new and original research findings, results of clinical studies, presentation of new and interesting clinical cases and recent evolutions. The journal will publish this information as up-to-date research articles, topical review articles, short communications, reports on interesting cases, as well as clinical studies commentaries. The foremost goal of this journal is thus to provide a platform to bridge the knowledge gap between physiological and pathological aspects of pig raising, to report on new developments and on important production management features, all within a science-based framework. This approach is unique at present in the porcine field worldwide.

We are interested in publishing articles of high quality and novelty, but we also consider research results of more general interest and clinical features to the readers. We believe that putting the pig at the center of this journal is unique and will serve both our favored animal species and our professions.

Being open access (http://www.porcinehealthmanagement.com/authors/instructions), all articles published in PHM by BioMed Central [4] are freely and globally accessible online and thus available to readers at no cost. Articles can thus reach a larger readership than with a subscription-based journal. Open access also allows rapid publication of reviews, articles and of new clinical data, with the opportunity to upload videos, photos, and datasets.

We believe that the open access format offers many advantages relative to traditional publication strategies, as articles will be published online very shortly after editorial acceptance which, markedly decreasing the time for distribution. All manuscripts will be independently reviewed by at least 2 experts in the field and by an Editor-in-Chief. The journal benefits from an editorial board of over 30 highly specialized members from 16 different countries. It is our aim to deliver a timely and fair peer review process. Authors of articles published in PHM retain the copyright of their articles and are free to reproduce and disseminate their work.

Among PHM’s inaugural articles are a review article on tail biting in modern pig production by Anna Valros and Mari Heinonen [5], a research article on serum haptoglobin dynamics and a commercial porcine circovirus type 2 vaccine by Lorenzo Fraile and colleagues [6], and a research article comparing lesions found in pigs under organic/free-range and conventional/indoor production conditions by Lis Alban and colleagues at the Danish Agriculture & Food Council [7].

We hope that you will enjoy the PHM journal and that you will consider it when deciding where to publish your work.

Sign up at http://www.porcinehealthmanagement.com/my/preferences to receive new article alerts from *Porcine Health Management.*
